# Coffee Silverskin as a Sustainable Alternative Filler for Plywood: Characterization and Performance Analysis

**DOI:** 10.3390/ma18071525

**Published:** 2025-03-28

**Authors:** Anita Wronka, Nidal Del Valle Raydan, Eduardo Robles, Grzegorz Kowaluk

**Affiliations:** 1 Institute of Wood Sciences and Furniture, Warsaw University of Life Sciences-SGGW, Nowoursynowska St. 159, 02-776 Warsaw, Poland; anita_wronka@sggw.edu.pl; 2 CNRS, Institute of Analytical and Physicochemical Sciences for the Environment and Materials (IPREM-UMR 5254), University of Pau and the Adour Region, 403 Rue Saint Pierre, 40004 Mont de Marsan, France; nidal.raydan@univ-pau.fr (N.D.V.R.); eduardo.robles@univ-pau.fr (E.R.)

**Keywords:** lignocellulosic materials, bio-based fillers, coffee industry by-products

## Abstract

Coffee silverskin, a by-product of coffee processing, was studied using microscopic (SEM), spectroscopic (FTIR), and thermogravimetric (TGA, DSC) methods to assess its use as a substitute filler in the manufacturing of plywood. TGA showed that the material was compatible with plywood hot pressing temperatures (140 °C) and that it was thermally stable up to 50 °C, with a notable decomposition event at 335 °C. Functional groups like hydroxyl and carbonyl were detected by FTIR analysis, indicating possible hydrogen bonds and chemical adaptability. DSC analysis confirmed structural alterations by highlighting endothermic processes associated with dehydration and an exothermic transition over 150 °C. Coffee silverskin substituted rye flour in plywood adhesive compositions at different concentrations (0%, 1%, 5%, 10%, and 20%). Due to the structural and chemical constraints of the filler, larger concentrations (10% and 20%) dramatically lowered bonding strength, whereas low silverskin amounts (1% and 5%) attained strengths equivalent to rye flour, reaching up to 5 N mm^−2^, according to internal bond strength tests. SEM images revealed smaller, more fragmented, and porous silverskin particles than larger, compact rye flour particles, which affected mechanical interlocking and adhesion. The findings point to coffee silverskin as an environmentally friendly and performance-balancing substitute for conventional fillers, especially at medium levels.

## 1. Introduction

Nestled in the heart of South America, Colombia is one of the most diverse countries in the world, boasting unparalleled richness in its natural landscapes and cultural heritage. This extraordinary diversity has made Colombia a global leader in exporting iconic products such as coffee and cocoa. These goods are emblematic of the nation’s identity and serve as vital pillars of its economy, driving growth and supporting countless livelihoods across the country. Coffee husk is one of the many waste products produced by the intricate process of making coffee. Large amounts of this byproduct are produced; an estimated 10 million tons of coffee husk trash are produced annually worldwide [1]. Coffee husk removal presents environmental issues, as poor handling can result in pollution, and it can draw pests and release dangerous toxins [1,2]. Sustainable methods for handling coffee husk waste are becoming more and more important. These include turning coffee husk into valuable products like biosorbents for environmental cleaning, renewable energy sources, and construction material components [3]. Coffee husk’s high organic matter content has made it a viable raw material for biofuel generation [3,4] and can be digested anaerobically to create biogas, a sustainable energy source [5]. Another valorization route is the production of bioethanol from coffee husk waste, which provides a sustainable way to lessen the negative consequences of fossil fuels, demonstrating its potential to support environmentally friendly and sustainable activities [6]. In addition, using coffee husk as an organic fertilizer can enhance soil with vital nutrients [7]. Coffee husk compost increases nitrogen (N) levels, and nitrogen is essential for plant growth, benefiting crops like maize and coffee seedlings by enhancing growth and biomass production [8,9,10]. It also boosts phosphorus (P) availability, which is crucial for root development and energy transfer, significantly improving soil phosphorus levels and crop yields when combined with other fertilizers [8,10]. Rich in potassium (K), it enhances water regulation and enzyme activation, improving plant health [9,10,11,12]. Additionally, coffee husk compost supplies micronutrients like iron (Fe), manganese (Mn), copper (Cu), and zinc (Zn), with composting enhancing their availability, further supporting soil fertility and plant nutrition [10,13].

According to a study, coffee husk ash (CHA) provides a sustainable substitute for conventional materials in the manufacturing of ceramics and soil stabilization [14,15]. Valuable components for culinary items, medicines, and other sectors can be derived through the treatment of coffee husk [3]. It has been demonstrated that producing coffee husk waste briquettes as an alternative fuel source is economically viable, with 7% being the ideal adhesive concentration for briquette manufacture. Coffee husks have a substantial amount of energy potential because of their high transformity and particular emerging values, which emphasize their efficiency in energy generation, making them a viable and sustainable alternative energy source minimizing reliance on non-renewable resources in thermoelectric systems, notwithstanding the environmental effects of their burning [16,17].

The use of coffee husks as a constituent in composite materials—often in place of wood or other conventional fillers—has been the subject of several investigations. Coffee husk has been utilized in biodegradable polymer composites, including polybutylene adipate terephthalate (PBAT) and polylactic acid (PLA). These composites’ improved mechanical qualities and fire resistance demonstrated their potential for environmentally benign uses [18,19]. Another example of coffee husk utilization is its incorporation into geopolymer matrices, demonstrating its potential in construction materials. These composites exhibited robust mechanical properties and thermal stability, making them suitable for various applications [20]. An example of innovative research on coffee husk utilization is a study of its use in alkali-activated bricks as a substitute for sand. The bricks were synthesized using ground granulated blast furnace slag (GGBFS), fly ash (FA), sand, and sodium silicate solution (SS), with sand partially replaced by waste coffee husk (WCH) at rates of 0–30% by volume. The study evaluated properties such as strength, density, water absorption, porosity, and efflorescence alongside structural and morphological characteristics using FTIR, XRD, TGA, and SEM analyses. The results demonstrated that incorporating WCH improved compressive strength up to 15.7 MPa and a density of 1509 kg m^−3^ at 30% WCH. However, water absorption and porosity increased due to the porous nature of WCH. Bricks with 30% WCH exhibited thermal stability and met the specific standards for good quality bricks, suggesting their feasibility for practical applications in the construction industry [21]. Another example of coffee husk fiber (CHF) application is as a reinforcing filler in thermoplastic composites, as CHF’s thermal characteristics and chemical makeup contrasted with wood fiber (WF). It was demonstrated that CHF has comparable thermal characteristics to WF and is mainly composed of cellulose, hemicellulose, and lignin. High-density polyethylene (HDPE) composites with a CHF content between 40 and 70% were created using melt processing and extrusion. Their mechanical behavior, water absorption, thermal performance, and processing characteristics were examined. Additionally, the impact of the coupling agent on maleated polyethylene (MAPE) was examined. According to experimental data, adding more CHF to the HDPE matrix improved the composites’ modulus and thermal characteristics but decreased their water resistance. The interfacial contact between the hydrophilic lignocellulosic fiber and the hydrophobic polymer matrix was considerably enhanced by adding 4% MAPE [22]. Coffee husk has also been utilized as reinforcement in other polymer composites. For example, unsaturated polyester composites reinforced with coffee husk powder and nano-silica showed better mechanical qualities and lower material costs [23]. Furthermore, coffee husk improved the mechanical qualities and water resilience of polypropylene (PP) composites [24,25].

Coffee husk exhibits various physical properties that make it suitable for multiple applications. The density of materials produced from coffee husks, such as particleboards and briquettes, meets specific standards, with smaller particles contributing to higher apparent density [26,27]. Coffee husk composites absorb more water, influencing their mechanical performance, especially in applications requiring moisture resistance [22,28]. Its thermal properties include enhanced insulation in ceramic products due to the formation of internal air chambers during combustion [28], as well as a high calorific value, making it a viable option for biofuels [29,30,31]. These properties are influenced by the chemical composition of coffee husk, consisting of cellulose, hemicellulose, lignin, and extractives [32,33]. Among the different parts of the coffee husk, the silverskin stands out due to its unique chemical composition, which contributes to its superior mechanical and durability properties. The silverskin contains a higher percentage of cellulose (23.8%) and a lower percentage of hemicellulose (16.7%) compared to the rest of the husk [34], and 30% lignin [35] (Table 1). This higher cellulose content improves the tensile strength of the material by providing a structural skeleton that enhances fiber-matrix adhesion [36]. As a result, it contributes to better overall stability and durability in composite materials, such as plywood. Additionally, the fine and uniform structure of silverskin allows for a more homogeneous distribution in the resin matrix, which is crucial for maintaining consistent mechanical performance [37]. The silverskin can be easily detached from the coffee beans during the roasting process. As the beans heat up and split, the silverskin loosens and peels off because of the physical alterations in the beans. This makes it possible to collect it as a by-product. The detached silverskin is usually gathered through techniques such as being blown away by air currents within the roasting machinery or manually extraction after roasting [38,39,40]. The mechanical properties of materials incorporating coffee husks depend on their composition and processing methods. Briquettes with smaller particles achieve greater compressive strength [27], while adding coffee husk ash to concrete can improve its compressive performance up to a specific replacement level [41]. When treated, coffee husk fibers enhance the tensile strength of composites, yielding notable improvements in this area [42,43]. Additionally, the flexural strength of coffee husk composites can be increased through coupling agents or supplementary fillers [22,43]. Coffee husk briquettes also display strong abrasion resistance, ensuring durability during storage and handling [27]. The individual properties and corresponding references have been summarized in Table 2 for better transparency.

Coffee husks, due to their properties, have the potential to be used as an environmentally friendly filler in plywood production. This potential is confirmed by using other alternative fillers that perform just as well in plywood technology as conventionally used rye flour. Examples of alternative raw materials in plywood technology include chestnut shells [44], hazelnut shells [45], buckwheat husk [46], beech bark [47], coffee grounds [48], and others. The present research aimed to evaluate the feasibility of using coffee husk as an alternative filler in plywood manufacturing.

## 2. Materials and Methods

### 2.1. Materials

The materials used for the test samples included the following: beech veneer (*Fagus sylvatica* L.), flat-cut with an average thickness of 0.62 mm. Powdered coffee silverskin (1/1 arabica/robusta) as a filler (Hive Roasters, Kotlarska St. 8, 64-610 Rogoźno k/Poznania, Poland), laboratory-ground on a Retsch SM 100 knife mill (Retsch GmbH, Haan, Germany) to a grain size of <0.125 mm; a moisture content of 2.4% ± 0.7%; coffee silverskin (CS) is composed mainly of dietary fiber, with up to 68.5% of mass content [49], a protein content between 7 and 22% [49], and a fat content around 1.5 and 2% [40]. Commercially available rye flour was used as a filler in the reference samples (REF); and a hardener consisting of 2% dry weight ammonium nitrate (NH_4_NO_3_) (CAS 6484-52-2, supplied by WARCHEM Sp. z o. o., Trakt Brzeski St. 167, 05-077 Zakręt, Poland) in an aqueous solution. Additionally, an industrial urea-formaldehyde resin (UF) (Silekol Sp. z o.o., Kędzierzyn-Koźle, Poland) with approximately 65% solid content and an average curing time of 98 s at 100 °C was used as an industrial reference. Type 3 water was also used in the formulation. The components were combined in the following nominal ratios: resin–hardener–filler–water, as shown in Table 2. The reference bonding mixture’s viscosity was approximately 440 mPa·s [50].

**Figure 1:**
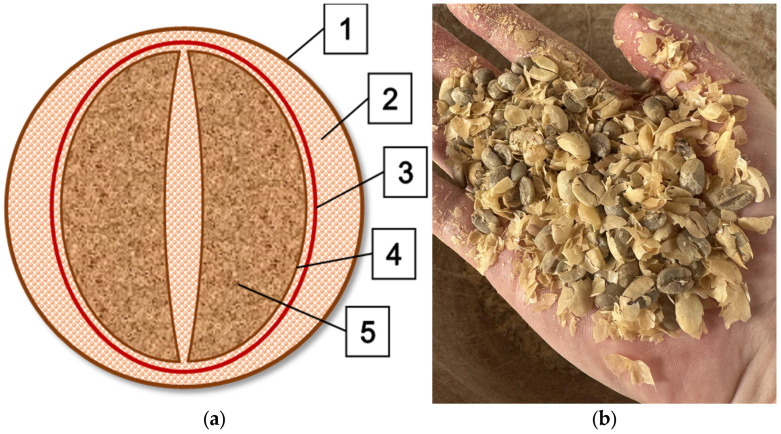
The scheme of the internal structure of coffee cherry (**a**) (1—skin, 2—pulp, 3—parchment, 4—silverskin, 5—bean) and silverskin separated before grain roast (**b**).

Figure 1 shows the internal structure of a coffee cherry (a), including (1) skin, (2) pulp, (3) parchment, (4) silverskin, and (5) the bean. Part (b) presents silverskin separated before grain roasting.

### 2.2. Composites Samples Preparation

Three-layer composites (plywood) were made using powdered silverskin as an alternative filler in the bonding process. Using a brush, the glue mixture of 180 g·m^−2^ was uniformly applied to the veneers. The samples were pressed through 5 min of high-temperature pressing (140 °C press temperature and 1 MPa maximum press unit pressure). After pressing, the samples were conditioned for 7 days at 20 °C and 65% ambient air humidity to stabilize the sample’s mass. The moisture content of the conditioned samples, measured by the kiln-dry method, was about 7.4% ± 0.4%. The varieties of the produced composites with their codes are presented in Table 3.

### 2.3. Characterization of the Manufactured Composites

The infrared spectra of coffee silverskin were recorded using a JASCO FT/IR-4700 Fourier Transform Infrared (FTIR) Spectrometer (JASCO International Co., Ltd., Tokyo, Japan) with a KBr beam splitter, working in ATR mode with a monolithic diamond crystal and a TGS detector; the spectral resolution was 0.964 cm^−1^. Plots correspond to an accumulation of 64 scans after treating the signal with log (1/T). Differential Scanning Calorimetry (DSC) tests were performed using a DSC3+ instrument (Mettler-Toledo, Viroflay, France) using hermetically closed aluminum crucibles. The measurements were conducted at a heating rate of 10 °C min^−1^. Samples weighing approximately 5 mg were analyzed. Thermogravimetric analysis (TGA) was performed using a Q500 instrument (TA Instruments, New Castle, DE, USA) under an inert nitrogen atmosphere with a flow rate of 40 mL·min^−1^). The temperature range analysis was 30–800 °C, with a heating rate of 10 °C·min^−1^. Samples weighing 5–10 mg were tested in titanium pans. A Quanta 200 FEG (FEI, Hillsboro, OR, USA) scanning electron microscope (SEM) was used to examine the surface morphology of the bonding lines containing different fillers under 30 kV accelerating voltage, using Backscattered Electron (BSE) Imaging. Internal bond strength tests followed standard [51] using computer-controlled universal testing equipment (Research and Development Center of the Wood-Based Panels Industry Sp. z o. o., Czarna Woda, Poland). For each tested variant, 12 samples with 50 mm × 50 mm dimensions were evaluated.

### 2.4. Statistical Analysis

Analysis of variance (ANOVA) and t-test calculations were used to test (α = 0.05) for significant differences between factors and levels using the IBM SPSS statistic base (IBM, SPSS 20, Armonk, NY, USA). A comparison of the means was performed when the ANOVA indicated a significant difference by applying the Duncan test. Where applicable, the mean values of the investigated features and the standard deviation values are presented on the plots as error bars.

## 3. Results and Discussion

### 3.1. FTIR, DSC, and TGA Characteristics

**Figure 2:**
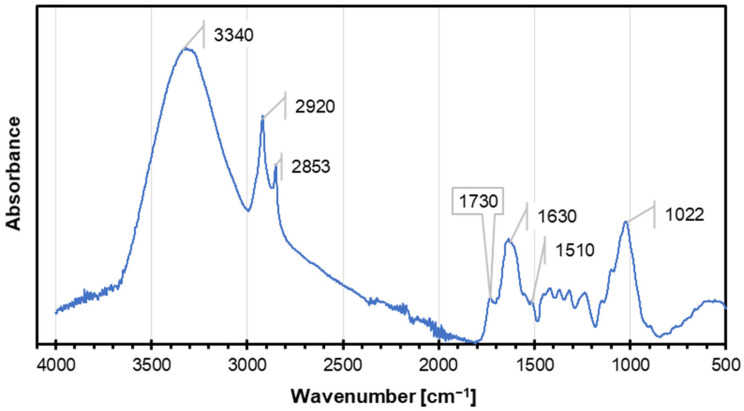
The FTIR spectra of coffee silverskin.

The FTIR spectrum presented in Figure 2 shows characteristic vibrations and their corresponding material properties. The broad and intense O-H stretching band (~3400–3600 cm^−1^) indicates the presence of hydroxyl groups (e.g., alcohols, phenols, or water). This suggests the material is likely hydrophilic with strong hydrogen bonding, which may enhance solubility, viscosity, and melting/boiling points. The strength of the hydrogen bonding can be inferred from the broad and intense nature of the O-H stretching band, which is typically associated with strong intermolecular hydrogen interactions. In comparison to weaker hydrogen bonds, which produce narrower and less intense bands, the broadness here indicates a stronger network of hydrogen bonds, likely due to the presence of multiple hydroxyl groups or water molecules interacting within the material. Bands in the C-H stretching region (~3100–2800 cm^−1^) correspond to C-H bonds in sp^2^ carbons (above 3000 cm^−1^, alkenes/aromatics) and sp^3^ (below 3000 cm^−1^, alkanes). These suggest the material is organic with hydrophobic regions that could contribute to amphiphilic behavior when combined with hydrophilic groups. Carbonyl groups (e.g., ketones, aldehydes, esters, or acids) produce strong bands in the C=O Stretching (~1750–1650 cm^−1^) range. The material may exhibit polar and reactive properties with good interaction potential for nucleophiles and moderate to high thermal stability. Bending vibrations of water molecules appear around 1630 cm^−1^ in FTIR spectra. This peak corresponds to the H-O-H bending mode, which arises from the deformation of the water molecule. The intensity of this band can vary depending on the hydrogen bonding environment. It is commonly observed in aqueous solutions and hydrated compounds. Vibrations in the C=C or aromatic structures (~1600–1500 cm^−1^) region suggest conjugated systems or aromatic rings, contributing to structural rigidity, UV-absorbing properties, and enhanced thermal stability. The fingerprint region (1500–500 cm^−1^) contains unique vibrations such as C-H bending, like in-plane bending, commonly found around 1300–1000 cm^−1^, C-O stretching—specified for carboxylic acids (COOH) appears around 1300–1200 cm^−1^, C-N stretching vibrations typically appearing in the FTIR spectra between 1500 and 1200 cm^−1^ for aliphatic amines and 1360 to 1250 cm^−1^ for aromatic amines, and out-of-plane bending for aromatic or alkene systems. The C-O stretching (~1022 cm^−1^) band indicates C-O bonds commonly found in alcohols, ethers, and esters. The material may be partially hydrophilic or contribute to the unique chemical reactivity of the system. Aromatic or carbonyl groups suggest good heat resistance, which is also confirmed by other studies [52], while aliphatic chains contribute to low thermal conductivity [53]. Strong hydrogen bonding and conjugated systems improve tensile strength, rigidity, and stability [54,55,56]. Aromatic systems and conjugated bonds enable UV absorption and potential semiconducting properties [57]. The material likely dissolves well in polar solvents (e.g., water, alcohols) if O-H or C=O groups are dominant or in nonpolar solvents (e.g., hexane) if aliphatic regions are present [58,59,60,61]. Functional groups like O-H, C=O, and C=C make the material chemically versatile and capable of esterification, polymerization, or hydrolytic biodegradability [62]. Comparing the FTIR spectra of the primary components, cellulose, hemicellulose, and lignin, however, shows that a large O-H stretching band at about 3330 cm^−1^ and a strong C-O stretching band at around 1050 cm^−1^ are typical characteristics of cellulose [63,64]. Though its spectra may have more intricate carbohydrate bands and changes in the C-O region, hemicellulose often displays comparable O-H stretching. Around 1600 cm^−1^, lignin exhibits significant aromatic C-H and C=C stretching. Its spectra lacks distinctive bands in the 1490–1530 cm^−1^ range [65]. This comparison indicates that although the material in question has some characteristics in common with cellulose (particularly the O-H and C-O stretching), it differs from lignin and hemicellulose in that it lacks strong aromatic bands and triple bonds, indicating a different chemical structure or modification.

**Figure 3:**
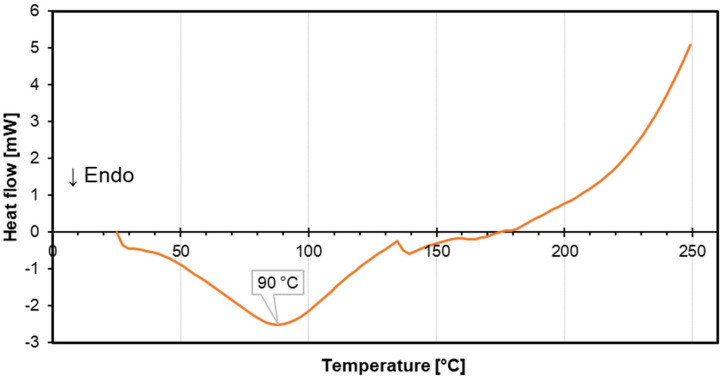
The DSC curve of coffee silverskin.

The results of the DSC analysis of coffee silverskin are presented in Figure 3. The heat flow decreases significantly, reaching a minimum of around 90 °C, indicating an endothermic event. This behavior is likely attributed to dehydration, as the release of moisture is standard in this temperature range for lignocellulosic materials [66,67,68]. The heat flow then begins to recover, followed by a plateau within the 90–150 °C temperature range, suggesting thermal stabilization after dehydration. A steep rise in heat flow is observed beyond 150 °C. This is characteristic of an exothermic event, such as crystallization, decomposition, or chemical reaction [69,70]. The sharp increase in heat flow continues beyond 250 °C, indicating sustained exothermic activity likely related to further decomposition of the material’s polysaccharides such as cellulose and hemicelluloses [71].

**Figure 4:**
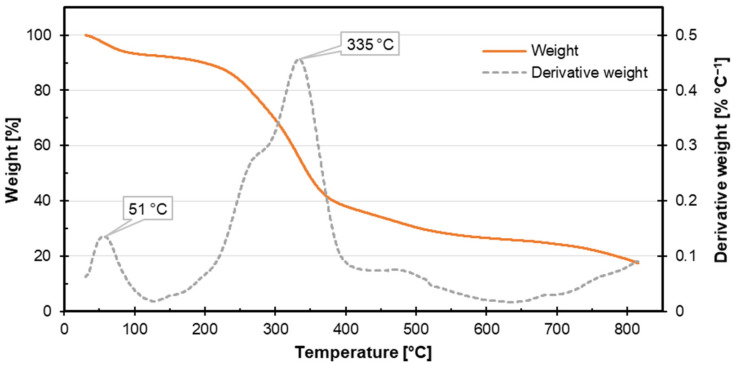
The TGA curve of coffee silverskin.

Thermogravimetric analysis (TGA) results for coffee silverskin are presented in Figure 4, showing the weight loss and its derivative as a function of temperature. At approximately 51 °C, an initial weight loss occurs due to the evaporation of moisture or volatile components, indicating that the material contains absorbed water or volatile impurities, a common characteristic of lignocellulosic byproducts [72]. A significant weight loss at 335 °C corresponds to the primary thermal decomposition of polysaccharides, where chemical bonds break, and gaseous byproducts are released. Beyond 335 °C, the weight loss progresses gradually up to 800 °C, reflecting the degradation of more thermally stable components, such as lignin, and the potential oxidation of remaining char residues [73]. The material demonstrates thermal stability up to 50 °C, with significant decomposition beginning at 335 °C, making this material suitable for applications below this temperature. The weight loss at lower temperatures indicates moisture sensitivity, which may influence the material’s physical and mechanical properties [74]. The residual weight at 800 °C likely represents inorganic components, such as ash or thermally stable char, providing insights into its composition and potential applications at high temperatures [75,76]. Based on the TGA results, the applied temperature of 140 °C during hot pressing for composite fabrication does not reach the coffee silverskin decomposition threshold, confirming its suitability as a sustainable alternative filler in plywood [71].

### 3.2. Internal Bond

**Figure 5:**
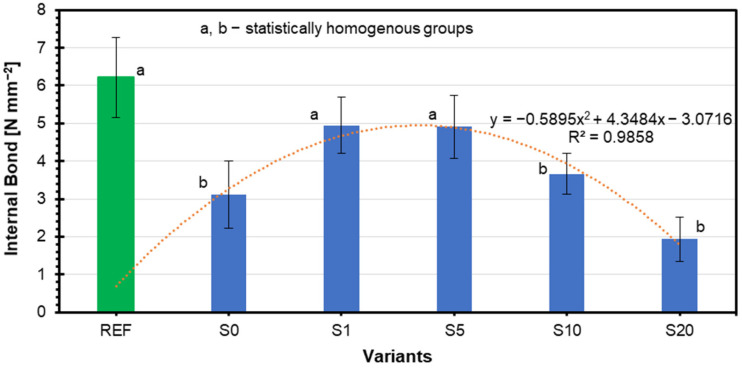
Internal bond of plywood with different amounts of coffee silverskin.

Figure 5 illustrates the internal bond strength of the various plywood samples. The REF plywood with rye flour filler achieves the maximum strength, reaching approximately 6 N·mm^−2^, as shown by the green bar. The other variants incorporate ground coffee silverskin as a filler in varying percentages, represented by S0, S1, S5, S10, and S20. Among these, S1 and S5 showed improved internal bond strength with 4 N·mm^−2^ and ~5 N·mm^−2^, respectively (statistical group “a”), while S0 exhibited the lowest bond strength of 1.5 N·mm^−2^ (statistically distinct group “b”). As the silverskin percentage increased, S10 and S20 gradually reduced bond strength, group “b”, with values decreasing to around 2–1.5 N·mm^−2^. The relationship between silverskin content and internal bond strength is strongly correlated and follows a quadratic relationship, as shown by the fitted equation: y = − 0.5895x^2^ + 4.3484x − 3.0716, with a determination coefficient of R^2^ = 0.9858. This correlation indicates a strong dependence of internal bond strength on silverskin content, where medium contents of silverskin result in optimal mechanical properties (S1 and S5), while higher contents (S10 and S20) reduce strength significantly. Individual characteristics of coffee silverskin, such as its chemical makeup—the amount of lignin, cellulose, and hemicellulose that can influence the composite’s stiffness and mechanical qualities—can impact the outcomes [77].

Additionally, components such as lipids, proteins, and phenolic substances can also weaken or enhance the links between polymers and fillers, reducing internal strength [78]. Similar results were obtained in producing plywood with coffee grounds, where increased coffee grounds content as a filler also led to a decline in mechanical properties [48]. The lower thermal stability of coffee silverskin may further contribute to the weakening of composite structures during hot pressing. For example, compared to traditional fillers like wood flour, food industry by-products such as coffee silverskin tend to have reduced thermal stability, affecting mechanical performance during fabrication [79].

### 3.3. SEM Image Analyses

**Figure 6:**
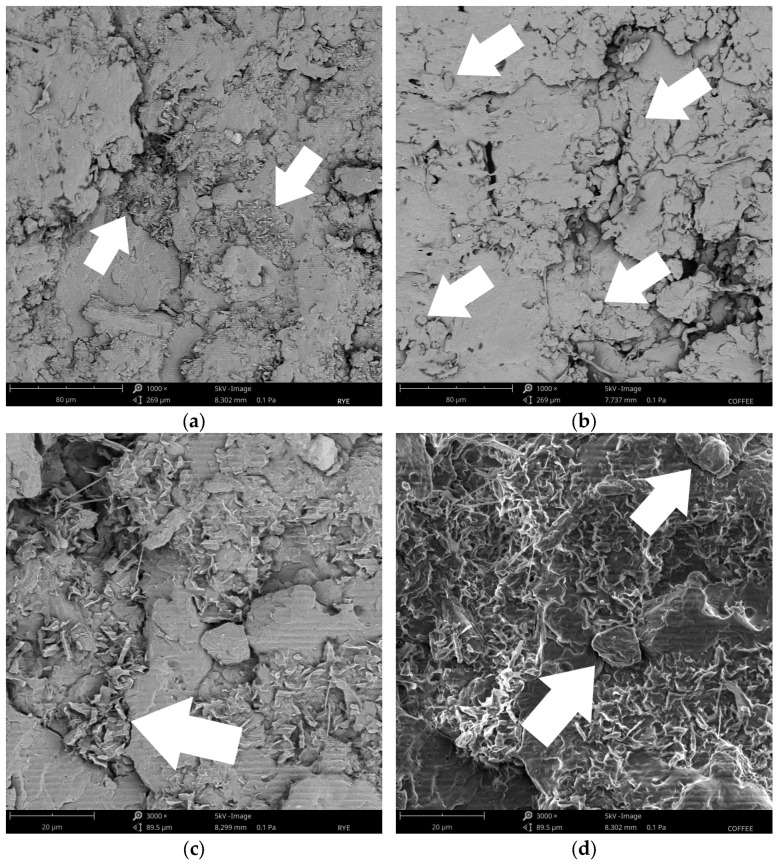
The SEM images of the bonding lines with various fillers: rye flour, 1000× (**a**) and 3000× (**c**) magnification; coffee silverskin, 1000× (**b**) and 3000× (**d**) magnification; the arrows indicate the filler particles.

Figure 6 shows SEM images of the bonding lines with various fillers, captured at magnifications of 1000× and 3000×. The bonding lines of plywood with rye flour and coffee silverskin fillers are clearly illustrated. At 1000× magnification, distinct rye flour particles can be observed as irregularly shaped structures dispersed along the bonding line (a). The arrows highlight the regions where the filler particles are embedded within the adhesive matrix. Gaps around the rye flour particles suggest limited compatibility with the adhesive matrix. At 3000× magnification (c), finer features of the rye flour particles reveal rough surfaces, which may contribute to mechanical interlocking. However, breaks in the bonding line remain visible, indicating that the adhesion is not uniform.

On the other hand, coffee silverskin particles exhibit smaller, more fragmented structures at 1000× magnification (b). These particles are spread more widely throughout the adhesive matrix but are also surrounded by gaps, as indicated by the arrows. This distribution suggests that achieving uniform bonding can be challenging. At 3000× magnification (d), the coffee silverskin particles show a fibrous and porous structure. While excessive porosity may result in weaker bonding regions, the particles’ irregularity and rough surfaces may improve mechanical interactions with the adhesive. The observed changes in mechanical performance in the plywood samples may be explained by the different interactions between the coffee silverskin fillers and rye flour, as indicated by the particle size, shape, and distribution variances. Rye flour particles are more extensive and less homogeneous due to their origin from finely ground rye grains. These particles are often compact and rounded, increasing the adhesive’s viscosity, ensuring more uniform distribution and improved adhesion [79,80]. The size and distribution of rye flour particles influence the adhesive’s viscosity, with smaller particles typically resulting in higher viscosity. This morphology can enhance the plywood’s mechanical properties and bonding strength [45,81]. In contrast, with its fibrous and fractured structure, coffee silverskin introduces a more irregular filler morphology, which may lead to changes in the plywood samples’ mechanical performance.

## 4. Conclusions

This study demonstrates that coffee silverskin can be utilized as an alternate filler in plywood manufacturing, partially substituting typically used rye flour. The presence of hydroxyl, aromatic, and carbonyl groups—which influence interactions with the adhesive matrix—was revealed by material characterization, including TGA, DSC, SEM, and FTIR analyses. Internal bond strength tests showed that mechanical properties improved when 1–5% coffee silverskin was added but deteriorated at higher concentrations (10–20%); compared to the denser rye flour, coffee silverskin’s fibrous and porous structure resulted in less homogeneous bonding, as observed in microscopic images of the fillers’ morphology. Thermal analysis confirmed the thermal stability of coffee silverskin during the pressing process, ensuring its suitability for such applications. Overall, the findings indicate that coffee silverskin can be used in moderate amounts as a sustainable and effective alternative to conventional fillers. This approach enhances resource efficiency and supports the wood industry in incorporating agricultural waste, thereby contributing to circular economy practices and reducing environmental impact.

## Figures and Tables

**Table 1:** Summary of the basic chemical composition of coffee silverskin.

Chemical Composition	Amount [%]	References
Cellulose	23.8	[34]
Hemicellulose	16.7	[34]
Lignin	30.0	[35]

**Table 2:** Individual properties of coffee husks.

Feature	Description	References
Density	Varies with particle size; higher for smaller particles	[26,27]
Water Absorption	Generally high, affecting mechanical properties	[22,28]
Thermal Properties	Good thermal insulation; high calorific value	[28,29,30,31]
Chemical Composition	Contains i.e., cellulose, hemicellulose, and lignin	[32,33]
Compressive Strength	Better with smaller particle size; enhanced in concrete	[27,41]
Tensile Strength	Enhanced with treated fibers in composites	[23,42]
Abrasion Resistance	Good; important for handling and storage	[27]
Flexural Strength	Improved with coupling agents or fillers	[22,43]

**Table 3:** The varieties of the produced composites with their codes.

Variant Code	Filler Type	Bonding Mass Composition * (by Weight)
REF	Rye flour	100:4:10:5
S0	Silverskin powder	100:4:0:5
S1		100:4:1:5
S5		100:4:5:5
S10		100:4:10:5
S20		100:4:20:5

* resin–hardener–filler–water.

## Data Availability

The original data presented in the study are openly available in RepOD at https://doi.org/10.18150/X8EKLS.
